# Integrative analysis of single-cell and bulk RNA sequencing reveals the oncogenic role of ANXA5 in gastric cancer and its association with drug resistance

**DOI:** 10.3389/fimmu.2025.1562395

**Published:** 2025-03-07

**Authors:** Denggang Chen, Peng Zhang, Li Gong, Hailang Wei, Guanghui Yu, Tingting Zhang, Chen Bai

**Affiliations:** ^1^ Department of General Surgery, Taihe Hospital, Hubei University of Medicine, Shiyan, China; ^2^ College of Life Sciences, South-Central Minzu University, Wuhan, China; ^3^ Department of Endocrinology, Taihe Hospital, Hubei University of Medicine, Shiyan, China; ^4^ Department of Clinical Oncology, Taihe Hospital, Hubei University of Medicine, Shiyan, China

**Keywords:** gastric cancer, single-cell RNA sequencing, prognostic model, immune infiltration, drug sensitivity

## Abstract

**Background:**

Gastric cancer (GC) remains a leading cause of cancer-related mortality, with over one million new cases and 769,000 deaths reported in 2020. Despite advancements in chemotherapy, surgery, and targeted therapies, delayed diagnosis due to overlooked early symptoms leads to poor prognosis.

**Methods:**

We integrated bulk RNA sequencing and single-cell RNA sequencing datasets from TCGA, GEO, and OMIX001073, employing normalization, batch effect correction, and dimensionality reduction methods to identify key cell populations associated with GC invasion and epithelial-mesenchymal transition (EMT), as well as analyze the tumor immune microenvironment.

**Results:**

Our analysis identified the MUC5AC+ malignant epithelial cell cluster as a significant player in GC invasion and EMT. Cluster 1, representing this cell population, exhibited higher invasion and EMT scores compared to other clusters. Survival analysis showed that high abundance in cluster 0 correlated with improved survival rates (P=0.012), whereas cluster 1 was associated with poorer outcomes (P=0.045). A prognostic model highlighted ANXA5 and GABARAPL2 as two critical genes upregulated in GC tumors. High-risk patients demonstrated increased immune cell infiltration and worse prognosic. Analysis of tumor mutation burden (TMB) indicated that patients with low TMB in the high-risk group had the worst prognosis. Wet-lab validation experiments confirmed the oncogenic role of ANXA5, showing its facilitation of cell proliferation, invasion, and migration while suppressing apoptosis.

**Conclusion:**

This study offers novel insights into the subpopulations of malignant epithelial cells in GC and their roles in tumor progression. It provides a prognostic model and potential therapeutic targets to combat GC, contributing crucial understanding to the fundamental mechanisms of drug resistance in gastrointestinal cancers.

## 1 Introduction

Gastric cancer (GC) represents a significant category of malignant tumors that endangers human health (1, 2). The global cancer report indicates that in 2020, more than one million new gastric cancer cases were diagnosed, leading to an estimated 769,000 fatalities, placing it fourth in the rankings of cancer-related mortality worldwide (3–5). The regions with the highest incidence of gastric cancer include Central and South America, Eastern Europe, and East Asia, most of which are developing countries (6–8). Due to the insidious onset of gastric cancer and its unobtrusive early symptoms, patients often miss the best treatment time when they seek medical attention (9–11). Although methods such as chemotherapy and surgery have to some extent extended the survival time of patients, and targeted therapy and immunotherapy have also shown therapeutic prospects in the treatment of gastric cancer, the effects of these treatments are still limited, and the prognosis for patients remains unfavorable (12–19). Faced with this clinical situation, exploring new diagnostic markers and therapeutic targets is particularly important (20).

Conventional bulk RNA sequencing (high-throughput transcriptome sequencing) technology is capable of targeting the average expression levels of numerous cellular transcripts within tissues (21, 22). However, this sequencing method overlooks the expression heterogeneity between various cells within the sample, concealing the existence and role of many special cell populations (23, 24). Advancements in sequencing technology led to the first report of single-cell RNA sequencing (scRNA-seq) in a study conducted in 2009, which significantly clarifies the transcriptomic variances among individual cells (25). At an unprecedented resolution, scRNA-seq sequences the gene expression information of individual cells, preserving the differences in transcriptomic information between different cells (26, 27). Single-cell transcriptomics can also identify cell-cell interactions based on the expression of cell receptors and ligands, and the formation of multimers, which gives it a significant advantage in studying the tumor microenvironment (28–32). At present, this technology has been utilized in studies focused on different kinds of tumors, such as breast cancer (33, 34), hepatocellular carcinoma (35, 36), lung cancer (37, 38), pancreatic cancer (39, 40), and melanoma (41, 42). In recent years, single-cell transcriptome sequencing has also been increasingly studied in GC (43, 44). Research based on gastric precancerous lesions has further confirmed the existence of spasmolytic polypeptide-expressing metaplasia (SPEM) at the single-cell level, revealing the potential transformation process from chief cells to neck cells and then to SPEM (45). The analysis of the immune microenvironment before and after chemotherapy for gastric cancer has pointed out that macrophages transform into M1 type after chemotherapy, and non-responders to chemotherapy exhibit T cells expressing LAG3, which may be related to drug resistance, revealing the reshaping of the tumor microenvironment by chemotherapy (46–49). However, there are currently fewer studies combining single-cell transcriptome sequencing with bulk data in gastric cancer, and the prognostic differences between gastric cancer at the single-cell level and high-throughput sequencing level are still unclear.

This research involved the integration of various existing bulk RNA-seq datasets alongside scRNA-seq data to delineate the single-cell architecture of gastric cancer tissue, emphasizing an examination of gene characteristics linked to the development and unfavorable outcomes of gastric cancer across diverse tissue types. We identified that the MUC5AC+ malignant epithelial cell cluster, represented by cluster 1, may be a key cell population in GC invasion and EMT. We also explored the tumor immune microenvironment and potential drug analysis. This study’s findings illuminate the role of specific subpopulations of malignant epithelial cells in the progression of gastric cancer, indicating potential therapeutic strategies and treatments to hinder the progression of this illness.

## 2 Materials and methods

### 2.1 Transcriptome data acquisition and processing

We acquired RNA expression profiles in conjunction with pertinent clinical information pertaining to gastric adenocarcinoma from The Cancer Genome Atlas (TCGA) database, specifically the TCGA-STAD dataset, which comprises 350 samples. This dataset served as the foundational training cohort for our research endeavors, providing essential data for the analysis and interpretation of the disease’s molecular characteristics and potential clinical implications. For the validation cohort, data was sourced from microarray datasets available in the Gene Expression Omnibus (GEO) database (GSE15460, n=248). In addition, the GSE55696 dataset and the GSE79973 dataset were also procured. The GSE55696 dataset encompasses 56 gastric tumor samples and 19 normal samples, while the GSE79973 dataset includes 10 gastric tumor samples and 10 normal gastric tissue samples. The data discussed earlier were converted into Transcripts Per Million (TPM) format and then log2 transformed for additional analysis. The techniques used for processing included: 1) Normalizing the data using the `normalizeBetweenArrays` function available in the `limma` package within R. 2) Eliminating batch effects from various datasets using the `Combat` function provided by the `sva` package in R. TCGA and GEO are public databases that allow us unrestricted access to patient data without the need for ethical approval.

### 2.2 Single-cell data acquisition and processing

Single-cell RNA sequencing data OMIX001073 was downloaded from the OMIX database. OMIX001073 comprises 23 primary gastric cancer samples. The R package “Seurat” was employed to perform single-cell standardization analysis using R software. The parameters for quality control that were set comprised a threshold for mitochondrial content of under 10%, whereas the defined upper and lower bounds for UMI counts and gene counts were 200-50,000 and 200-8,000, correspondingly. Data normalization was conducted using the “NormalizeData” function from the “Seurat” package, followed by the application of “FindVariableFeatures” and “ScaleData” functions to identify 2,000 highly variable genes for transformation to mitigate cell cycle influences. To address batch effects, the “harmony” function was utilized. Dimensionality reduction was performed using UMAP and tSNE, while clustering was conducted through the Louvain algorithm. The “FindAllMarkers” function was used to evaluate differential gene expression across clusters or cell types, following the thresholds of p < 0.25 and expression proportion greater than 0.1.

### 2.3 Cell annotation analysis

This research characterized epithelial cells by employing markers such as “EPCAM,” “KRT18,” “KRT19,” and “CDH1,” while fibroblasts were identified using “DCN,” “THY1,” “COL1A1,” and “COL1A2.” Endothelial cells were characterized by the markers “PECAM1,” “CLDN5,” “FLT1,” and “RAMP2.” For T cells, the markers used were “CD3D,” “CD3E,” “D3G,” and “TRAC.” In the case of NK cells, identification was achieved with “NKG7,” “GNLY,” “NCAM1,” and “KLRD1.” B cells were identified through the use of “CD79A,” “IGHM,” and “IGHG3,” while mast cells were recognized via “KIT,” “MS4A2,” and “GATA2.” Following this, the specified markers facilitated the categorization and clustering of diverse cell types to investigate tumor heterogeneity, with visualization techniques including UMAP, tSNE, bar graphs, and heatmaps.

### 2.4 Epithelial cell subgroup analysis and copy number variation analysis

An analysis of subgroups was performed on the specified epithelial cells, from which the CNV results were gathered for the sake of clustering. Subsequently, endothelial cells were utilized as a benchmark to pinpoint other cells for the analysis of malignancy and to evaluate the CNV scores of the clusters generated by the epithelial cells.

### 2.5 Pseudotime analysis and transcriptional factor analysis of epithelial cells

The “Seurat” single-cell standardization workflow employed the monocle2 package to conduct pseudotime analysis on subgroups of epithelial cells, aiming to clarify the processes of cellular differentiation. Concurrently, SCENIC software was employed to analyze transcription factors within epithelial cell subgroups. The RcisTarget software package was used to identify transcription factor binding motifs that showed increased expression according to the gene list, whereas the AUCell software was applied to assess the activity of each group of regulators throughout all cell types.

### 2.6 Cell communication analysis

The CellChat software package is employed to assess potential cell-cell communication. Functions utilized include “identify OverExpressed Genes,” “identify overExpressed Interaction,” “ProjectData,” “computeCommunoProb,” “filterCommunication,” and “computecommunoProbPathway.” These functions serve to identify possible interactions between ligands and receptors. Ultimately, the “aggregateNet” function is utilized to create a network for cell-to-cell communication.

### 2.7 Differential gene analysis and enrichment analysis and ssGSEA

Differential gene analysis was performed on gastric cancer tissue samples and normal gastric tissue samples sourced from the GEO and TCGA datasets, using the “limma” and “clusterProfiler” packages in R, with a significance threshold set at P<0.05. Furthermore, single-sample gene set enrichment analysis (ssGSEA) was utilized to compute the enrichment scores of the gene sets within the samples, which allowed for the determination of risk scores for each patient.

### 2.8 Establishment of GC related risk signatures

This research determined the overlap of differentially expressed genes derived from epithelial cells, utilizing both the GEO and TCGA datasets to isolate gastric cancer-related genes (GCRGs). Following this, univariate Cox analysis was conducted to associate GCRGs with clinical prognosis data of gastric cancer, thereby identifying genes linked to prognosis in gastric cancer. Then, the prognostic model was established by Lasso regression method and verified by ROC curve through the “timeROC” package. The model enhances the selection of prognostic genes used to calculate the risk score for each gastric cancer patient. The TCGA cohort patients were divided into high-risk and low-risk categories according to the median score, and the predictive accuracy of the model was assessed.

### 2.9 Prediction of immunotherapy response, tumor immune infiltration analysis, and tumor immunophenotype analysis

The risk model scores for each dataset, including GSE35640 (melanoma), GSE91061 (melanoma), IMvigor210 (urothelial carcinoma, UC), and GSE126044 (cancer, NSCLC), were compiled to evaluate the association between immunotherapy responses and varying risk levels. This process aims to validate the efficacy of the predictive model applied in this study. Following this, the immune infiltration levels in the TCGA STAD cohort were assessed using six methods: CIBERSORT, quanTIseq, MCPcounter, xCell, EPIC, and Estimate. A heatmap showcased the relative presence of stromal cells, immune cells, and tumor cells. Furthermore, the Tumor Immunophenotype (TIP) analysis was conducted at this website: http://biocc.hrbmu.edu.cn/TIP.

### 2.10 Drug sensitivity analysis and TMB analysis

This study employs a detailed collection of half-maximal inhibitory concentration (IC50) values for commonly utilized chemotherapeutic agents available in the “oncoCpredict” package for R, aiming to assess the relationship between risk scores and drug sensitivity. Additionally, the Wilcoxon rank-sum test is utilized to investigate variations in IC50 values between the two risk groups. For the analysis of Tumor Mutational Burden (TMB), cancer mutation information is obtained from the TCGA GDC database and processed with the maftools package.

### 2.11 Cell culture and transfection

Validation through experiments was conducted utilizing various human gastric cancer cell lines, including HGC-27, MKN-45, SNU-1, NUGC-3, AGS, along with the human gastric epithelial cell line GES-1. These cell lines were supplied by the Cell Bank of the Chinese Academy of Sciences. The HGC-27, MKN-45, SNU-1, and NUGC-3 cell lines were cultured in Dulbecco’s Modified Eagle Medium (DMEM, HyClone, USA), while the AGS and GES-1 lines were maintained in Roswell Park Memorial Institute 1640 (RPMI-1640, HyClone, USA). All media were supplemented with 10% fetal bovine serum (FBS, KeyGEN, China) and 1% penicillin-streptomycin (Procell, China) to ensure optimal cell viability and minimize the likelihood of bacterial contamination. The cell cultures were maintained at 37°C in a humid environment with a CO_2_ level of 5%. Daily monitoring was performed to verify that the cells were in the ideal logarithmic growth phase, and they were passaged every 24 hours. For the transfection protocols, we utilized siRNA (Sangon, China) targeting ANXA5 to reduce its expression levels, using a si-negative control for comparison. Cells were removed from the culture flasks via trypsin (KeyGEN, China), subsequently washed with PBS twice, and subjected to centrifugation. After being resuspended in fresh culture medium, the cell concentration was determined. Approximately 2×10^4^ cells were plated per well in 6-well plates with 2 mL of complete medium. Once the cells attached to the plates, siRNA and Lipofectamine™ 3000 (Invitrogen, USA) were combined at a predetermined ratio, allowed to sit at room temperature for 15 minutes, and then subjected to low-speed centrifugation for 1 minute. The mixture was introduced to the cells in each well. Media change was conducted 4 hours following transfection, with subsequent experiments carried out 48 hours later.

### 2.12 Total RNA extraction and RT-qPCR

RT-qPCR was performed to assess the mRNA levels of ANXA5. After forty-eight hours post-transfection, the culture medium was removed, and the cells were gently washed with PBS before undergoing trypsin digestion. Following several PBS washes and low-speed centrifugation, we collected the cell pellet. In accordance with the manufacturer’s guidelines, an adequate volume of Trizol reagent (Takara, Japan) was used to lyse the cells. After maintaining the solution on ice for 10 minutes, we gradually introduced 200 µL of chloroform (SINOPHARM, China), along with equal volumes of isopropanol (SINOPHARM, China) and anhydrous ethanol (SINOPHARM, China). With each addition of the organic solvents, the mixture was permitted to incubate at low temperatures, followed by centrifugation to remove the organic solvent. Ultimately, all organic solvents were disposed of, and the RNA pellet was allowed to dry in a laminar flow hood for a duration of 40 minutes. Subsequently, the RNA was dissolved in 20 µL of DEPC-treated water, and its concentration was assessed using a Nanodrop 2000 (Thermo, USA). Following the manufacturer’s guidelines, the RNA was treated with the PrimeScript RT Reagent Kit (Takara, Japan) to eliminate the genomic DNA, facilitating the reverse transcription process required for cDNA synthesis. For the quantitative PCR (qPCR), cDNA samples were prepared utilizing the SYBR GreenER Supermix (Takara, Japan) kit, making certain that every 18 µL reaction contained 2 µL of cDNA. The 7500 Real-Time PCR System (Thermo Fisher Scientific, USA) was employed to carry out real-time quantitative PCR. We analyzed the relative expression of ANXA5 using the 2^–ΔΔCt^ method, with β-actin serving as the normalization control.

### 2.13 CCK-8 assay

Forty-eight hours after transfection, trypsin (KeyGEN, China) was used to dissociate the cells, which were then evenly distributed in complete medium. According to the findings from the cell count, a total of 5,000 cells were added to each well of a 96-well plate. To ensure accuracy, all experimental groups were performed in triplicate. Once the cells had adhered to the surface, the CCK-8 reagent (KeyGEN, China) was combined with complete medium, reaching a total volume of 200 µL for each well, following the manufacturer’s guidelines. This mixture was quickly added to the wells, and the plate was then covered with aluminum foil to shield it from light. After an incubation period of 1.5 hours, absorbance was recorded at 450 nm with a microplate reader. This entire process was repeated at 24, 48, 72, and 96-hour intervals.

### 2.14 Flow cytometry

Cell apoptosis was evaluated through flow cytometry. The cells were dissociated using trypsin without EDTA (Beyotime, China), and the resulting cell pellet was obtained by centrifugation at 2,000 RPM for a duration of 5 minutes. Afterwards, the cells were rinsed three times with cold PBS (4°C) and then resuspended in tubes for flow cytometry. Following the guidelines provided by the manufacturer, sufficient amounts of propidium iodide (PI, Biosharp, China) and FITC-Annexin V (FITC, Biosharp, China) were added to every sample. The cells underwent a 15-minute incubation in the dark at a temperature of 37°C before being analyzed using a flow cytometer. Each experimental group was performed in triplicate.

### 2.15 Total protein extraction and western blotting

Protein lysates were produced by combining RIPA buffer (Beyotime, China) with a protease inhibitor (Beyotime, China) in a 100:1 ratio, following the instructions provided by the manufacturer. This mixture was then transferred to a centrifuge tube that contained the cell pellet, ensuring that the cells were well resuspended. Subsequently, sonication was performed at an amplitude of 40% for 1 second per pulse, with this process being repeated three times. The lysates were kept on ice for 30 minutes, during which vortexing and centrifugation were conducted every 10 minutes. Following this, the lysate underwent centrifugation at 10,000 RPM for 15 minutes at 4°C, and the supernatant was carefully collected for the measurement of protein concentration. This supernatant was combined with sample loading buffer according to the determined protein concentration, heated to 95°C for 5 minutes, and then allowed to cool to room temperature. Protein samples, amounting to 20 µg per lane, were applied to a 10% SDS-PAGE gel, which was operated at 100V and then transferred to a PVDF membrane with a pore size of 0.45 µM (IPVH00010, Millipore). The membrane received a blocking treatment with QuickBlock™ Blocking Buffer (Beyotime, China) for a duration of 10 minutes. Following this, it was washed three times with TBST containing 0.1% Tween-20 and incubated overnight at 4°C with primary antibodies. After a 16-hour incubation period, the membrane was washed three more times with TBST and then incubated at room temperature for 1.5 hours with HRP-conjugated secondary antibodies. Visualization of the protein bands was achieved through enhanced chemiluminescence (ECL, Beyotime, China). Primary antibodies for β-actin, ANXA5, and the secondary antibody were sourced from Proteintech Group, Inc.

### 2.16 Transwell assay

Matrigel (Corning, USA) was diluted at a proportion of 1:7, and 45 µL was introduced into each chamber (Corning, USA). Subsequently, the chambers were positioned in a sterile biosafety cabinet for a drying period of 36 hours to confirm sterility. For the migration assays, no Matrigel was applied to the chambers. A total of 700 µL of complete medium was dispensed into each well of a 24-well plate. Following a transfection period of 48 hours, the cells were dissociated using trypsin and subsequently resuspended in a medium lacking FBS. The concentration of cells was assessed, and 20,000 cells were added to each chamber along with 170 µL of medium without FBS. These chambers were placed in a 24-well plate and incubated for 24 hours in a cell culture incubator, ensuring that the complete medium covered the bottom of each chamber. After the incubation period was completed, the medium was thrown away, and the cells were carefully rinsed with PBS. Next, the cells were fixed for 30 minutes using 4% paraformaldehyde, then washed again, and any non-invading cells were carefully removed with a cotton swab. Subsequently, the cells were stained using a solution of 0.1% crystal violet for 20 minutes and rinsed three times with PBS. Photographs of the stained cells were taken and cell counts were conducted under a microscope. This procedure was replicated to validate the results’ reliability.

### 2.17 Statistical analysis

Data management, statistical analysis, and graphical representations were conducted utilizing R software version 4.1.3. We employed Pearson’s correlation coefficient to evaluate the connection between two continuous variables. The chi-square test was employed for comparing categorical variables, whereas the Wilcoxon rank-sum test or t-tests were used for continuous variables. In the context of survival analysis, the survival package was employed to carry out Cox regression analysis and construct Kaplan-Meier survival curves. A p value < 0.05 was considered statistically significant (* p < 0.05; ** p < 0.01; *** p < 0.001; **** p < 0.0001).

## 3 Results

### 3.1 Single-cell expression atlas of STAD

**Figure 1:**
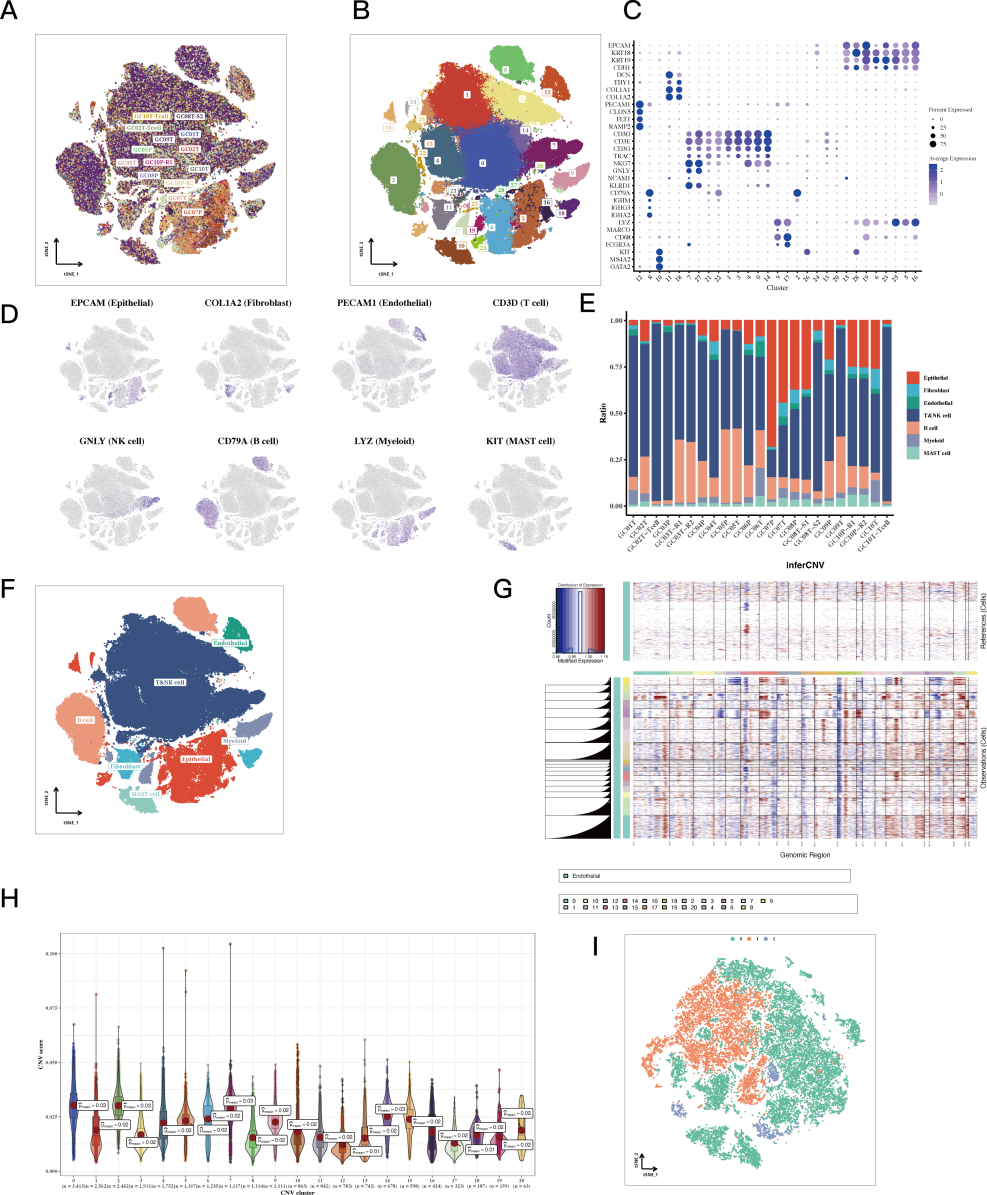
Single-cell classification results of STAD. **(A, B)** tSNE plots colored by sample and cluster assignment. **(C)** Correlation plots based on marker gene expression matrices for each cluster. **(D)** tSNE plots of marker gene expression for each cluster. **(E)** Bar plots showing the composition of cell types in each sample. **(F)** tSNE plots of cell annotation results. **(G)** CNV heatmap results of epithelial cells with endothelial cells as reference. The copy number variation on all chromosomes of each cell subgroup was significantly higher than that of the reference group. **(H)** Violin plots of CNV scores for epithelial cell sub-clusters. The 12th and 17th subgroups had the lowest average CNV score of 0.01. **(I)** tSNE plots of epithelial cells classified by CNV score.

The study included 23 distinct single-cell samples from gastric cancer, with each displaying a fairly uniform cell distribution, indicating minimal impact from batch effects (refer to **Figure 1A**). Utilizing the tSNE algorithm, every cell was meticulously classified into 29 separate clusters (see **Figure 1B**). An extensive bubble plot illustrated the expression profiles of specific marker genes linked to the 23 cell clusters (illustrated in **Figure 1C**). Furthermore, a graph depicting gene expression pertinent to cell type identification was provided (shown in **Figure 1D**). A plot (see **Figure 1E**) subsequently illustrated the distribution of fibroblasts, endothelial cells, T cells, NK cells, B cells, mast cells, and epithelial cells among the 23 samples of gastric cancer. Significantly, the presence of different cell types was illustrated in **Figure 1F**, which includes epithelial cells, T cells, and fibroblasts. Additionally, by utilizing inferred CNV, the status of copy number variation (CNV) for every chromosome was elucidated, indicating that epithelial cells generally exhibited a greater CNV compared to endothelial cells in most cases (**Figure 1G**). Notably, substantial decreases in copy numbers on chromosome 6 were observed in almost all tumor cells. To evaluate the variations in CNV scores among various clusters, particular attention was given to those with increased copy number variations—specifically clusters 0, 2, 7, and 14—along with clusters that exhibited lower copy numbers, including clusters 13 and 17 (**Figure 1H**). Following the application of tSNE for dimensionality reduction, the epithelial cell clusters were divided into three distinct subclusters: subcluster 0, subcluster 1, and subcluster 2 (see **Figure 1I**).

### 3.2 Trajectory analysis and cell communication analysis of epithelial cells

**Figure 2:**
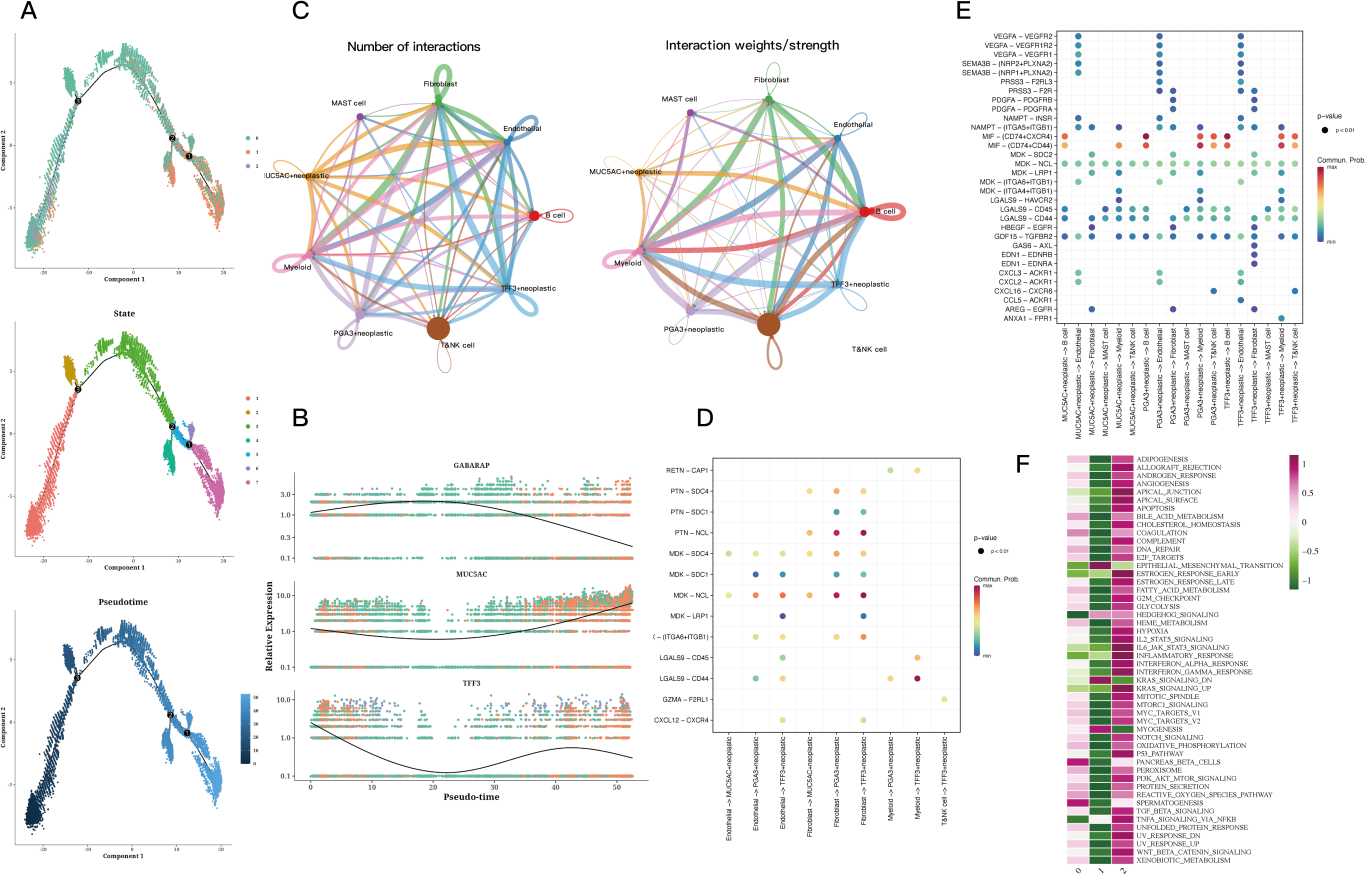
Trajectory and cell communication analysis results of epithelial cells. **(A)** Differentiation trajectory, pseudotime distribution, pseudotime cell clusters, and the proportion of each cluster among all epithelial cells. The cells in 0 and 2cluster of CNV score were mainly concentrated in the early and middle pseudotime, while the cells in 1cluster were mainly concentrated in the late pseudotime. State 1 is mainly concentrated in the early proposed time, state 2 and 3 are mainly concentrated in the middle proposed time, and the remaining states are concentrated in the late proposed time. The expression of GABARAP increased first and then decreased, while the expression of MUC5AC decreased first and then increased in pseudo-time. In addition, the expression of TFF3 decreased first and then fluctuated. **(B)** Relative expression of GABARAP, MUC5AC, and TFF3 across pseudotime. **(C)** Quantity and strength of cell communication between PGA3+ tumors, MUC5AC+ tumors, TFF3+ tumors, and other cell types. **(D)** Bubble plots of ligand-receptor actions of PGA3+ tumors, MUC5AC+ tumors, TFF3+ tumors on different cell types. **(E)** Bubble plots of different types of cells acting on PGA3+ tumors, MUC5AC+ tumors, TFF3+ tumors. **(F)** Enrichment analysis of the three clusters.

Pseudotime analysis indicated that subtype cluster 0 is prevalent during the early stage, while subtype cluster 2 occupies a transitional phase, and subtype cluster 1 is positioned at the later stage (**Figure 2A**). Subsequently, the expression levels of GABARAP, MUC5AC, and TFF3 were presented, as their alterations were particularly significant, offering insights into the temporal fluctuations in gene expression (**Figure 2B**). The quantity and intensity of cellular interactions among PGA3+ tumor cells in cluster 0, MUC5AC+ tumor cells from cluster 1, TFF3+ tumor cells associated with cluster 2, and other diverse cell types were then illustrated (**Figure 2C**). Cell-type-specific ligand-receptor interactions with the three marked tumor cells within the tissue were analyzed. Notably, we found that PGA3^+^ tumor cells engage with other cell types through the MDK-NCL receptor-ligand pair. Similarly, MUC5AC^+^ tumor cells establish connections with other cells via the MDK-NCL receptor-ligand pair (**Figures 2D, E**). Ultimately, the enrichment analysis demonstrated that cluster 0 is enriched in nearly all pathways, highlighting its significance across various biological processes. In contrast, cluster 0 is primarily enriched in spermatogenesis and pancreatic beta cells, while cluster 1 specifically enriches in EMT and myogenesis pathways (**Figure 2F**).

### 3.3 Transcriptional factor analysis of epithelial cells

**Figure 3:**
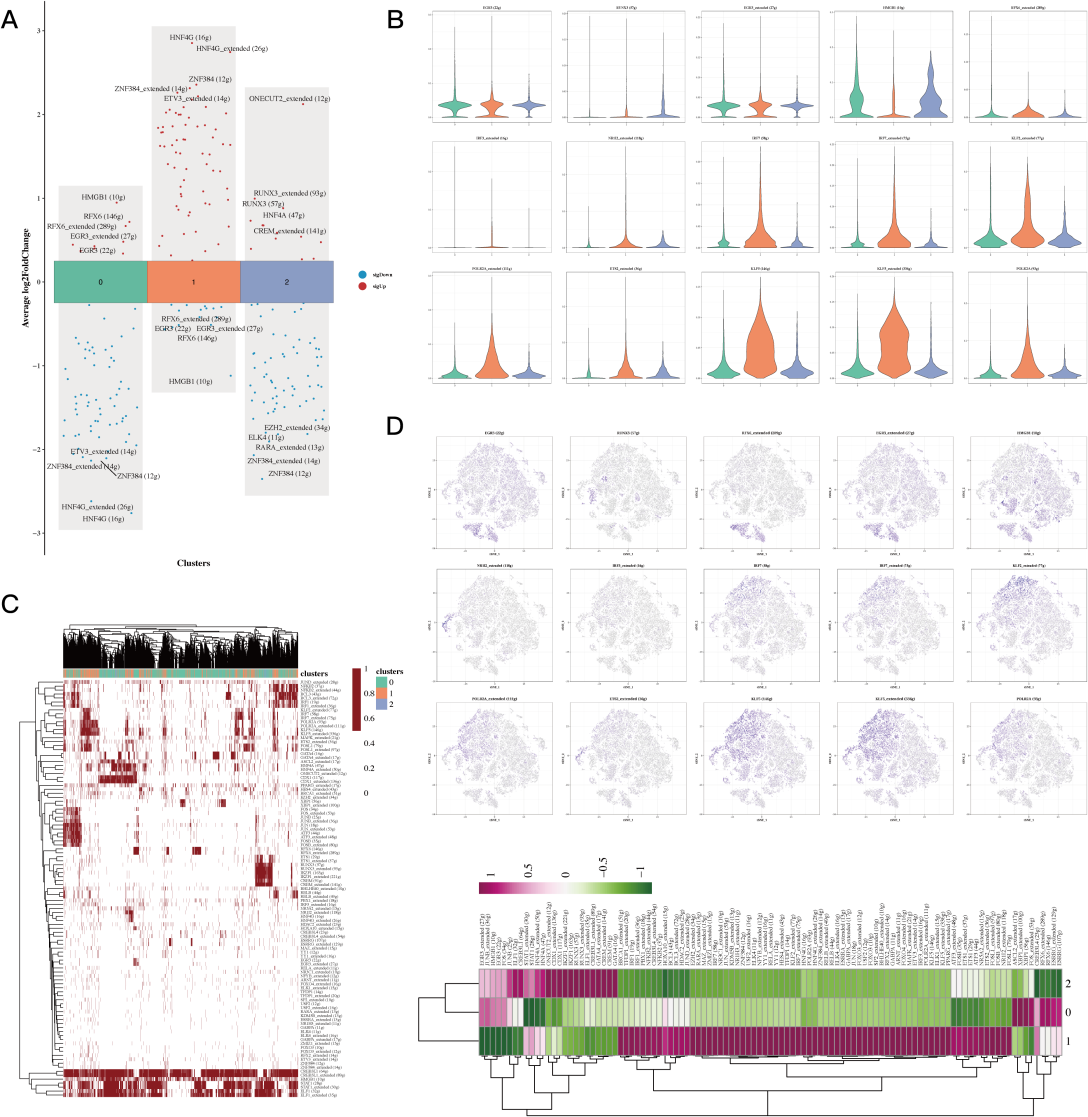
Transcriptional factor analysis results of epithelial cells. **(A)** Volcano plots showing the top 5 highly and lowly expressed genes in each cluster. **(B)** Violin plots and UMAP plots of expression for 5 genes in each cluster. **(C, D)** Heatmaps showing the distribution of gene regulatory elements in different clusters.

The differential analysis identified the five transcription factors exhibiting the highest expression levels along with the five that showed the lowest expression in every cell cluster (**Figure 3A**). The tSNE and violin plots depicted the expression patterns of these genes, highlighting their regulatory functions within each cluster (**Figure 3B**). Additionally, heatmaps were generated to depict how differential gene regulatory elements are distributed across the three cell clusters (**Figures 3C, D**).

### 3.4 Functional analysis of aggressive and EMT

**Figure 4:**
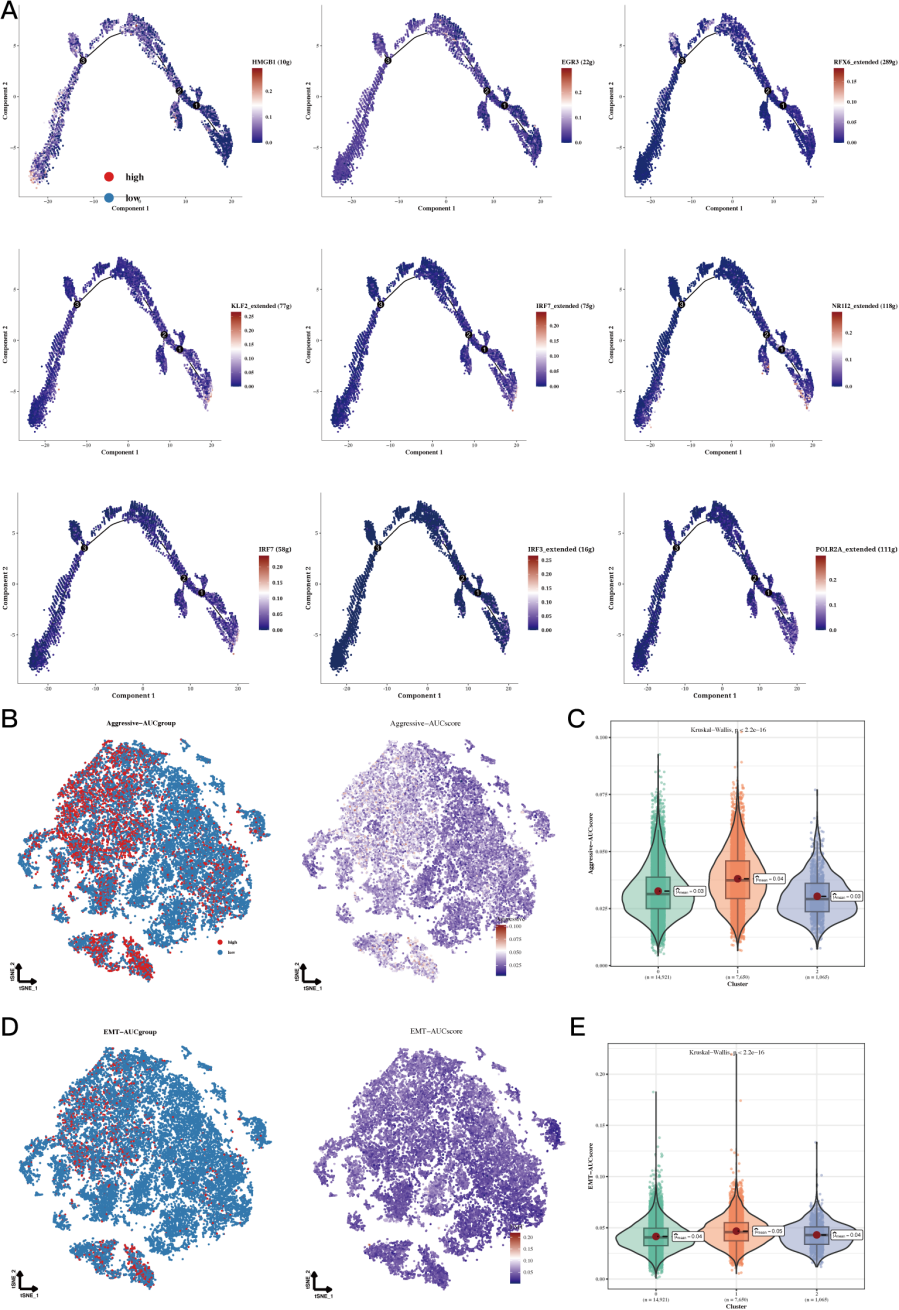
Functional analysis results of Aggressive and EMT. **(A)** Cell trajectory analysis of different regulatory clusters. The expression of HMGB1 decreased in the pseudo time, EGR3, RFX6_extended increased and then decreased in the pseudo time. KLF2_extended, IRF7_extended, NR1I2_extended, IRF7, IRF3_extended and POLR2A_extended increased in pseudotime. **(B, C)** TSNE plots and violin plots showing the invasive levels of the 3 clusters. **(D, E)** TSNE plots and violin plots of EMT levels in the 3 clusters.

Pseudotime analysis focused on highly specific transcription factors indicated that EGR3, HMGB1, and RFX6 show increased expression in cluster 0, whereas IRF7, KLF2, and NR1I2 demonstrate upregulation in cluster 1. Additionally, elevated levels of IRF3, IRF7, and POLR2A were observed in cluster 2 (**Figure 4A**). Invasion assays revealed that subgroup 1, in comparison to other cellular subgroups, displayed significantly greater invasion scores, suggesting that tumor cells in this cluster possess enhanced invasive abilities (**Figures 4B, C**). Epithelial-mesenchymal transition (EMT) scoring revealed significant differences between cluster 1 and clusters 0 and 2, with cluster 0 showing markedly higher EMT scores than clusters 1 and 2 (**Figures 4D, E**). The variations suggest that gastric epithelial cells categorized under subgroup 1 demonstrate enhanced migratory capabilities, which may be linked to a heightened likelihood of metastasis (**Figure 4A**, P<0.05).

### 3.5 Prognostic model establishment and evaluation

**Figure 5:**
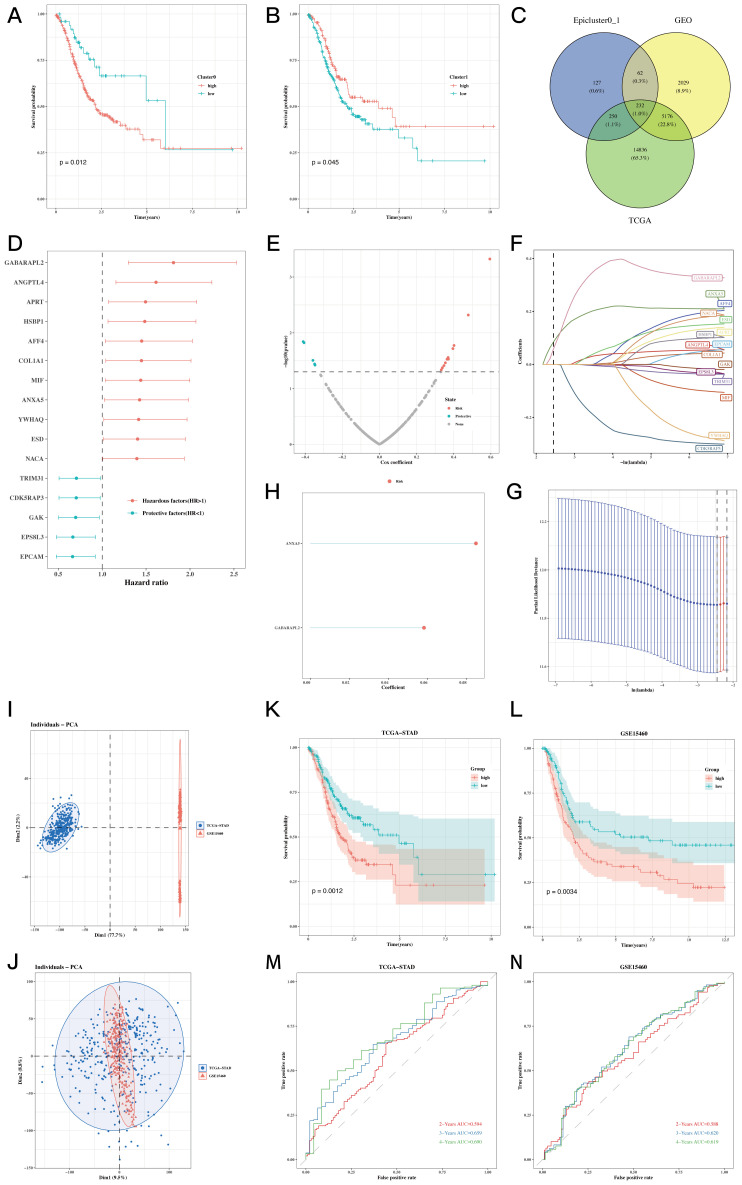
Prognostic model establishment and evaluation results. **(A, B)** Impact of cluster 0 and 1 abundance on survival. **(C)** Venn diagram showing the intersection genes of Epicluster0_1 with GEO and TCGA cohorts. **(D)** Forest plot showing the results of univariate COX analysis. **(E)** Volcano plot showing upregulated and downregulated differential genes in the cohort. **(F, G)** LASSO regression for screening important prognostic genes. **(H)** Distribution of model gene signature values. **(I, J)** Detectable batch effects in TCGA and GEO data cohorts, with mitigation of batch effects to ensure coordinated data integration. **(K-N)** Survival differences between high-risk and low-risk groups in the TCGA cohort and GEO datasets and their respective time ROC curves.

By employing the ssGSEA method, we assessed the presence of signature genes within clusters 0 and 1 in samples from TCGA-STAD. The analysis of survival suggested that a higher abundance of genes in cluster 0 was associated with enhanced survival rates (**Figure 5A**, P=0.012), while the results for cluster 1 showed the opposite trend (**Figure 5B**, P=0.045). A Venn diagram displayed 232 marker genes related to epithelial cell subgroups identified from the TCGA database, GEO data, and differential genes between clusters 0 and 1 (**Figure 5C**). Subsequent univariate COX analysis with the aforementioned genes and TCGA-STAD survival data identified 16 prognostic genes, and a forest plot exhibited 5 protective factors and 11 risk factors (**Figure 5D**, P<0.05). Lasso regression analysis in machine learning based on the collection of prognostic-related genes opened a prognostic model containing 2 genes (**Figures 5E-H**). Batch effects were removed for the TCGA data and GEO datasets to facilitate subsequent model validation (**Figures 5I, J**). External validation was then conducted using TCGA and GEO data, and survival analysis showed that the prognosis of the high-risk group in the TCGA cohort was significantly poorer than the low-risk group (**Figure 5K**, P=0.0012). This finding was strongly corroborated within the GEO cohort (**Figure 5L**, P=0.0034). Finally, the AUC of ROC curve at 2years, 3years and 4years were all greater than 0.58, indicating that this model has a good predictive ability for the prognosis of gastric cancer patients (**Figures 5M, N**).

### 3.6 Immune infiltration analysis

**Figure 6:**
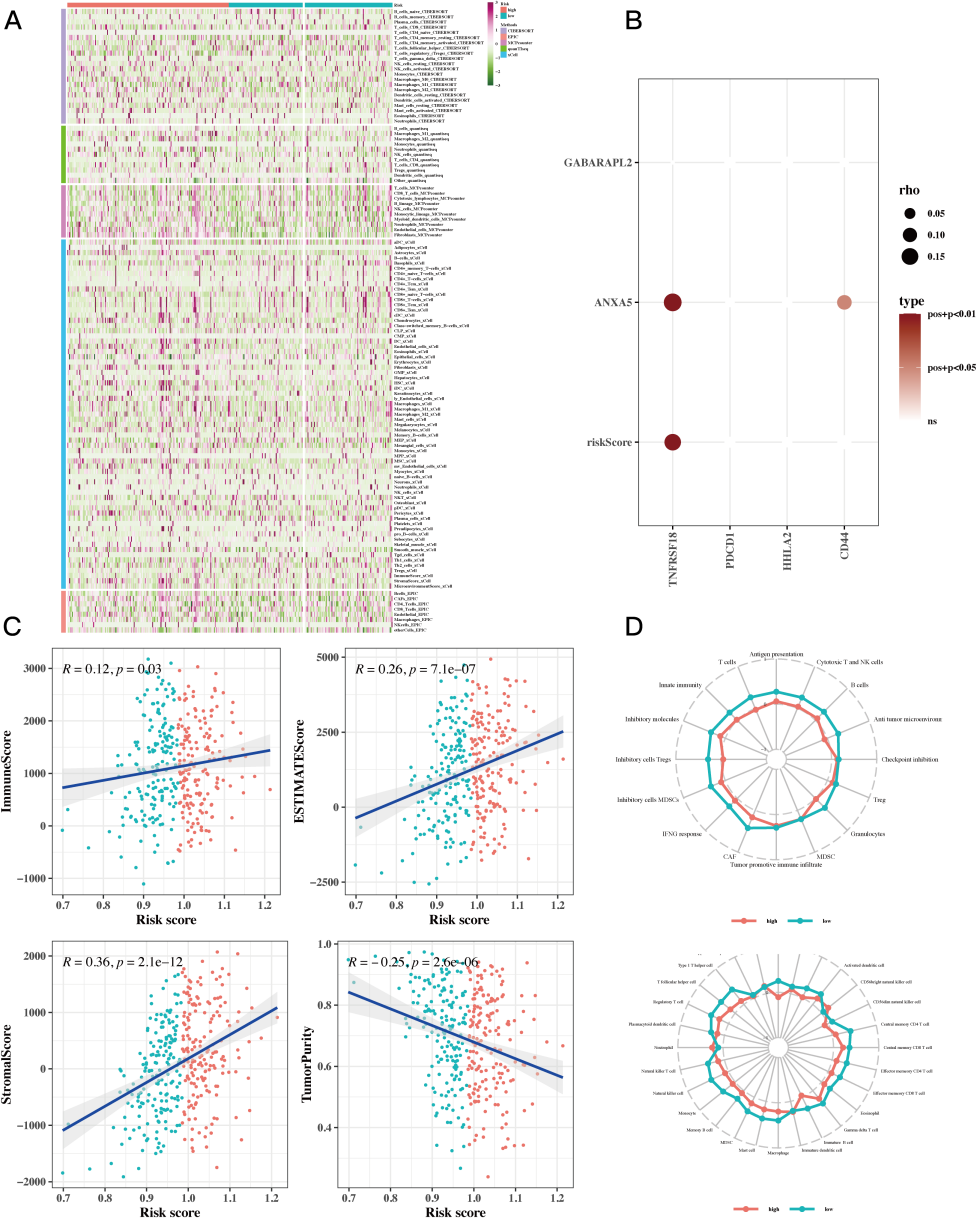
Immune infiltration analysis results. **(A)** Heatmap showing differences in immune cell infiltration between two risk groups assessed by five algorithms. **(B)** Bubble plots showing the correlation between riskScore and some model genes and immune checkpoint expressions. **(C)** Scatter plots illustrating the correlation between risk scores and stromal scores, immune scores, ESTIMATE scores, and tumor purity, revealing the intricate interconnections within the tumor microenvironment. **(D)** ssGSEA enrichment analysis showing the comparison of immune cell infiltration and immune-related pathways associated with risk values between high-risk and low-risk groups in a radar chart.

To assess the levels of immune cell infiltration in gastric cancer (GC) patients categorized as high-risk compared to those considered low-risk, we utilized tools such as CIBERSORT, quanTIseq, MCPcounter, xCell, EPIC, and Estimate. The analysis revealed a notably greater abundance of immune cells in the high-risk group. Among them, CD8+ T cells, B cells, and natural killer cells (NK cells) showed increased infiltration in high-risk groups. These cells play a key role in anti-tumor immunity. CD8+ T cells can directly kill tumor cells. Although B cells are primarily involved in humoral immunity, they may also be involved in the regulation of the tumor microenvironment. NK cells can recognize and kill tumor cells without prior sensitization (**Figure 6A**). A bubble plot revealed notable associations between TNFRSF18 and risk scores, along with prognostic model genes (**Figure 6B**, P<0.05). Investigating the relationship between immune infiltration levels indicated a meaningful positive correlation between risk scores and immune scores, while a negative correlation was found between risk scores and tumor purity (**Figure 6C**). ssGSEA findings suggested that in the low-risk cohort, immune cell infiltration displayed a positive relationship with risk values. Furthermore, this low-risk group exhibited stronger associations across various immune-related pathways (**Figure 6D**).

### 3.7 TMB analysis revealed that the combination of the low TMB group and the high-risk group had the poorest prognosis

**Figure 7:**
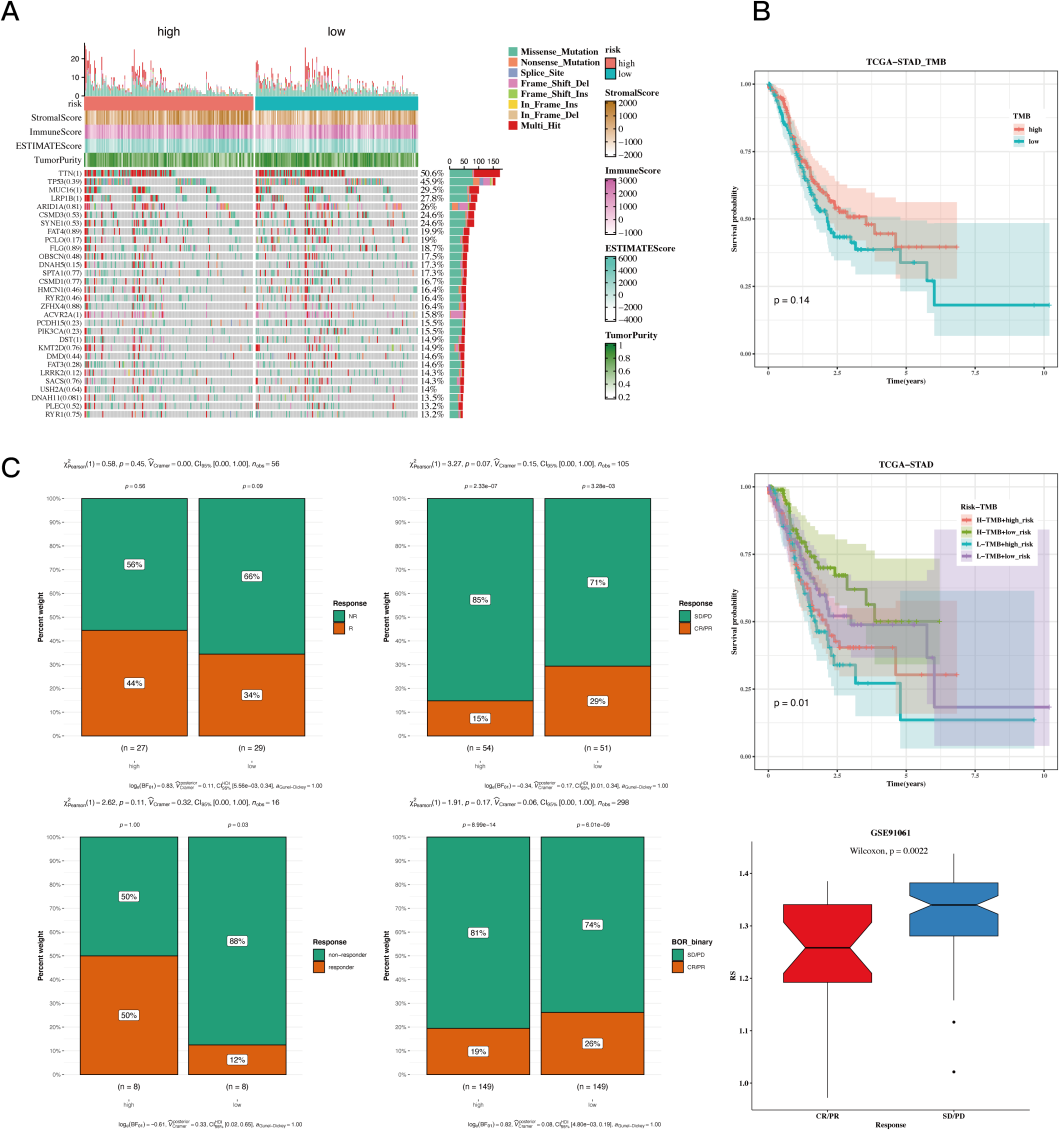
TMB and immunotherapy analysis results. **(A)** Waterfall plot depicting differences in mutated genes in high-risk and low-risk populations. Mutation rates are shown, with genes sorted by mutation frequency. **(B)** Survival curves showing differences in survival among different subgroups. **(C)** Composition analysis of various immunotherapy datasets to assess the association between risk values and immunotherapy response.

The waterfall plot displayed typical gene mutations within both the high-risk and low-risk groups, showing that the genes with the highest mutation frequency were TTN, TP53, MUC16, LRP1B, and ARID1A, with no significant variation in mutation profiles between the two groups. Notably, the heatmap revealed no significant difference in tumor mutational burden (TMB) across these groups (**Figure 7A**). However, when patients were divided based on TMB levels, it became evident that individuals in the low TMB category had a worse prognosic in comparison to those in the high TMB group; specifically, the cohort identified by both low TMB and high risk showed the least favorable prognosis (**Figure 7B**). Additionally, in several different tumor groups receiving immunotherapy, including GSE91061, IMvigor210, GSE126044, and GSE35640, comparative studies indicated that a larger percentage of patients classified as low-risk exhibited notably higher responder rates compared to those categorized as high-risk. This supports earlier findings that the low-risk cohort displayed more powerful associations with various immune-related pathways. Finally, we evaluated risk scores in responders versus non-responders within the GSE91061 dataset, showing that non-responders exhibited significantly higher risk scores than responders (**Figure 7C**).

### 3.8 Enrichment and drug sensitivity analysis

**Figure 8:**
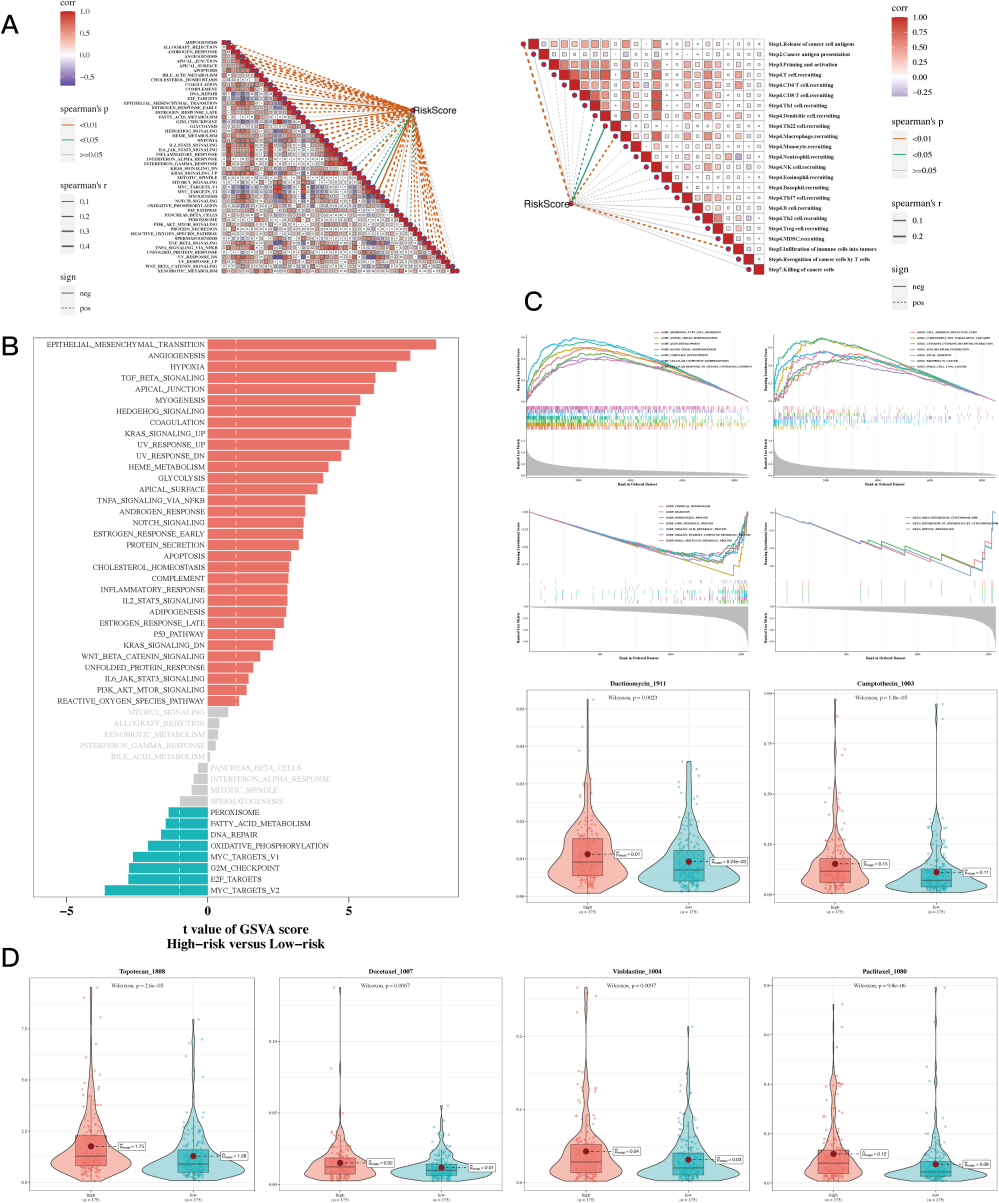
Enrichment and drug sensitivity analysis results. **(A)** The relationship between risk score and tumor immune cycle steps and biomarker gene sets. **(B)** GSVA enrichment analysis showed that the standard gene set was enriched between high-risk and low-risk groups. **(C)** GSEA enrichment analysis showing differential enrichment of various genes in GO and KEGG pathways between different risk groups. **(D)** Box plots comparing sensitivity to six chemotherapy drugs between high-risk and low-risk groups.

The thorough correlation analysis involving risk scores, the cancer immune cycle, and various gene sets showed a noteworthy negative correlation between risk scores and a majority of the components in the cancer immune cycle. Conversely, a positive correlation was noted between risk scores and most oncogenic pathways (**Figure 8A**). Additionally, gene enrichment analysis revealed that hypoxia and epithelial-mesenchymal transition (EMT) are primarily enriched in the high-risk gastric cancer (GC) population (**Figure 8B**). GSEA unveiled distinct enrichments in GO and KEGG pathways (**Figure 8C**). Drug sensitivity analysis identified “Dactinomycin_1911”, “Docetaxel_1007”, “Vinblastine_1004”, “Paclitaxel_1080”, “Camptothecin_1003”, “Topotecan_1808” as potential effective drugs for GC treatment (**Figure 8D**, P<0.05).

### 3.9 Validation analysis of model genes

**Figure 9:**
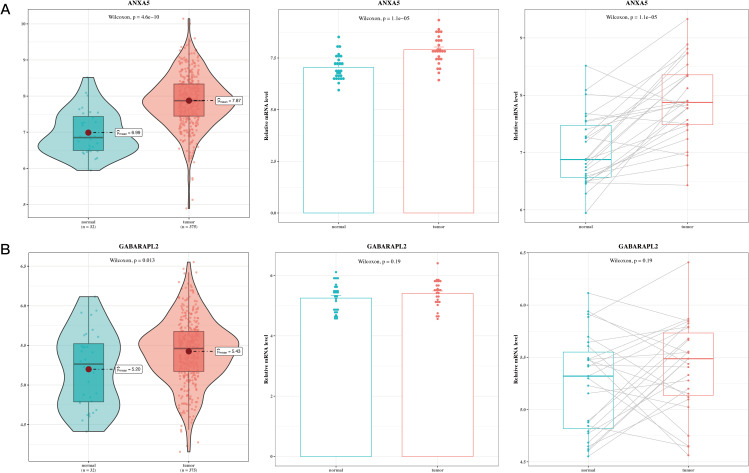
Validation analysis results of model genes. **(A)** Box plot of differential expression of ANXA5 in TCGA tumor and normal tissues; gene expression of ANXA5 in 27 paired cancer and adjacent samples. **(B)** Box plot of differential expression of GABARAPL2 in TCGA tumor and normal tissues; gene expression of GABARAPL2 in 27 paired cancer and adjacent samples.

Ultimately, this research performed a correlation analysis on the two significant genes within the prognostic model utilizing TCGA-STAD data. This included comparisons of normal gastric tissues with gastric tumor tissues, as well as paired evaluations of gastric tumor tissues against adjacent non-cancerous tissues. The analysis revealed that ANXA5 and GABARAPL2 were upregulated in gastric cancer tumor tissues (**Figures 9A, B**, P<0.05).

### 3.10 ANXA5 promotes gastric cancer progression

**Figure 10:**
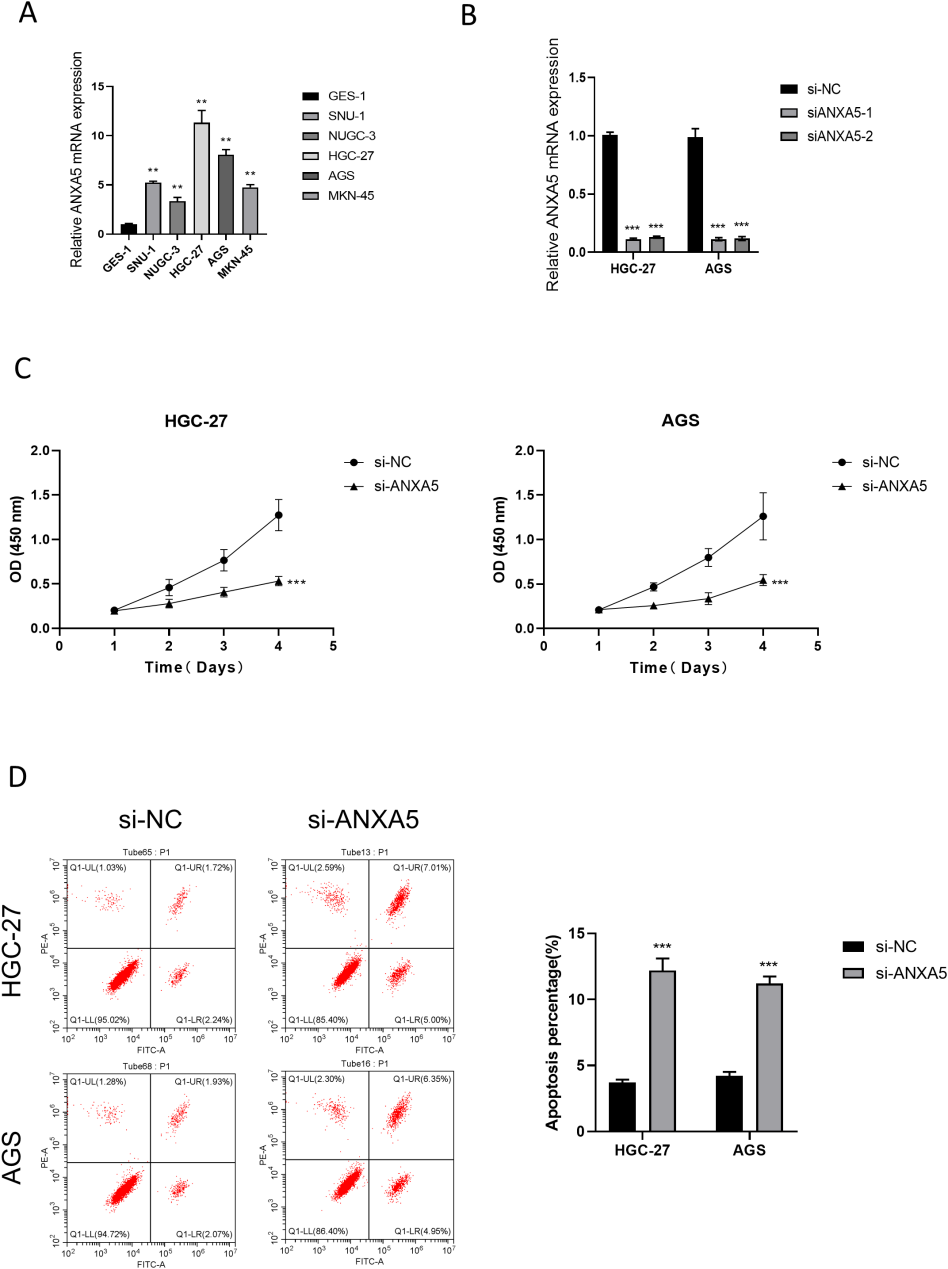
The relationship between ANXA5 and gastric cancer was investigated through a series of experiments. **(A)** Real-time quantitative reverse transcription PCR (qRT-PCR) analysis shows that ANXA5 gene expression is significantly elevated in gastric cancer cell lines, with higher expression observed in the HGC-27 and AGS cell lines. This experiment demonstrates differential ANXA5 expression across various cell lines. **(B)** Real-time quantitative reverse transcription PCR (qRT-PCR) analysis reveals that siRNA-mediated knockdown of ANXA5 expression results in a significant decrease in ANXA5 gene expression in both HGC-27 and AGS cell lines. This experiment confirms successful silencing of ANXA5 expression by siRNA. **(C)** CCK-8 cell viability assay shows a significant reduction in cell viability in both HGC-27 and AGS cell lines following siRNA-mediated ANXA5 knockdown. This experiment suggests that ANXA5 promotes cancer cell proliferation. **(D)** Flow cytometry analysis demonstrates a significant increase in apoptotic cell populations in both HGC-27 and AGS cell lines following siRNA-mediated knockdown of ANXA5. This experiment indicates that ANXA5 inhibits cancer cell apoptosis.

**Figure 11:**
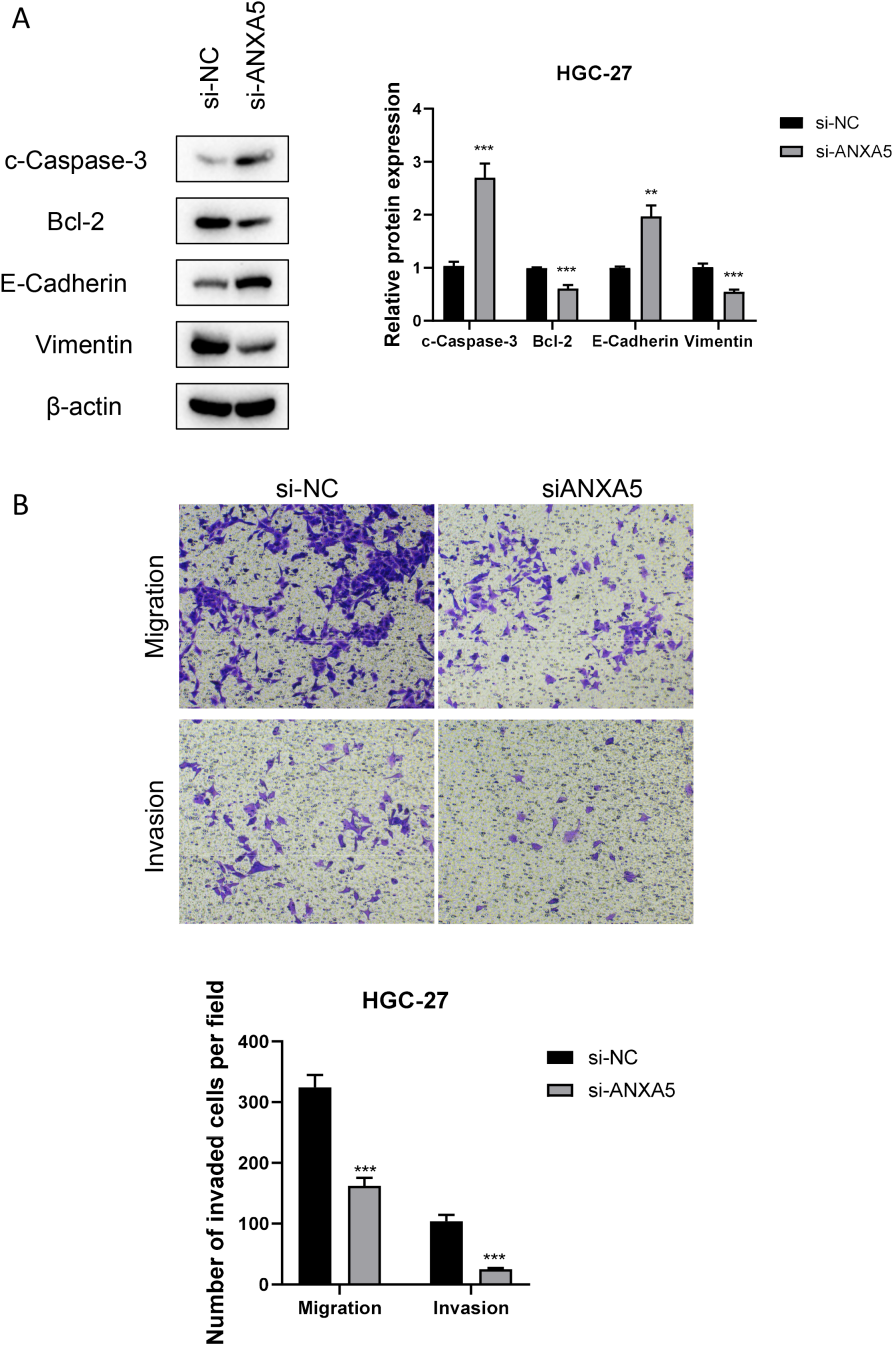
Impact of ANXA5 Knockdown on Apoptosis, Migration, and Invasion in HGC-27 Gastric Cancer Cells. **(A)** Western blot analysis reveals changes in the expression of relevant proteins (c-Caspase-3, Bcl-2, E-Cadherin, Vimentin) in the HGC-27 cell line following siRNA-mediated ANXA5 knockdown. This experiment shows that silencing of ANXA5 increases the expression of c-Caspase-3 and E-Cadherin, while decreasing the expression of Bcl-2 and Vimentin. **(B)** Transwell migration and invasion assays demonstrate a significant reduction in migration and invasion capabilities of HGC-27 cells following siRNA-mediated ANXA5 knockdown. This experiment indicates that ANXA5 promotes cancer cell migration and invasion.

At the cellular level, gastric cancer cell lines exhibited notably higher ANXA5 expression compared to normal gastric epithelial cell lines. The HGC-27 and AGS cell lines specifically exhibited the highest levels of ANXA5, which were chosen for additional experimentation (P<0.01, **Figure 10A**). To investigate the functional roles of ANXA5, we employed siRNA to diminish the expression of ANXA5 in two distinct human gastric cancer cell lines, achieving a significant knockdown efficiency as evidenced by RT-qPCR (P<0.001, **Figure 10B**). Results from the CCK-8 assay indicated that the downregulation of ANXA5 led to a notable reduction in cell viability in both HGC-27 and AGS cells. This implies that ANXA5 is involved in facilitating the proliferation of gastric cancer cells (P<0.001, **Figure 10C**). Additionally, flow cytometry analysis showed a significant rise in the rates of apoptosis in these cell lines after the knockdown of ANXA5 (P<0.001, **Figure 10D**). Additionally, Western blot analysis supported these outcomes, indicating that the absence of ANXA5 resulted in an elevation of the pro-apoptotic protein c-Caspase-3 and the adhesion protein E-Cadherin; conversely, levels of the anti-apoptotic protein Bcl-2 and the mesenchymal marker Vimentin diminished, affirming the molecular function of ANXA5 (P<0.01, **Figure 11A**). Finally, Transwell assays revealed that the suppression of ANXA5 expression significantly impaired the migration and invasion capabilities of HGC-27 cells, suggesting that ANXA5 enhances the migratory and invasive characteristics of gastric cancer cells (P<0.001, **Figure 11B**). Collectively, these findings indicate that ANXA5 possesses a pro-tumorigenic function in gastric cancer, facilitating cell proliferation, invasion, and migration while inhibiting apoptosis.

## 4 Discussion

Gastric cancer represents a common type of malignant tumor located within the gastrointestinal tract, generally associated with an unfavorable prognosis (50, 51). Our extensive research utilizing both bulk RNA sequencing and single-cell RNA sequencing data has illuminated the complex landscape of gastric cancer, uncovering the detailed diversity of cellular populations and their contributions to tumor progression and outcomes. The integration of these high-throughput sequencing approaches has allowed us to dissect the tumor microenvironment with unprecedented resolution, identifying key cellular clusters and molecular signatures associated with GC invasion, EMT, and patient survival.

The identification of the MUC5AC+ malignant epithelial cell cluster as a potential driver of GC invasion and EMT underscores the importance of targeted therapeutic strategies (52, 53). Studies have successfully constructed prognostic models capable of predicting the survival time of GC patients based on multiple genes including MUC5AC, and have indicated its potential involvement in the pathway of apoptosis (54). Avanbakht’s research indicated that the mRNA expression levels of MUC5AC in GC tissues were significantly lower when compared to non-cancerous tissues. The reduced levels of MUC5AC were linked to more aggressive features of the tumor, including TNM staging, histological classification, and lymph node metastasis. The study concluded that MUC5AC downregulation could be associated with both the advancement of the disease and a poorer prognosis for GC (55). However, other studies have used *in vitro* models of gastric mucosa to investigate the impact of exogenously expressed Helicobacter pylori virulence factor CagA on the expression of MUC5AC. It was found that the H. pylori-derived virulence factor CagA can increase the expression of MUC5AC (56). Thus, the specific mechanism of MUC5AC in GC remains unclear. This study, taking a scRNA-seq perspective to re-examine its potential role in GC, will provide new insights into GC research. This cell population, represented by cluster 1, may serve as a critical therapeutic target, warranting further investigation into its specific vulnerabilities and potential for intervention.

This study successfully constructed a model capable of predicting the prognosis of GC patients based on ANXA5 and GABARAPL2. Research has found that ANXA5 is associated with tumor-associated macrophages and has preliminarily verified through immunohistochemistry and angiogenesis experiments that ANXA5 has the role of predicting the survival time of GC patients (57). Higher expression levels of ANXA5 were also identified as promoting tumor angiogenesis in GC. Interestingly, however, another study highlighted ANXA5’s function as a gastric cancer tumor suppressor gene that inhibits the ERK signaling pathway, promising a supportive anticancer drug (58). This difference is worthy of further study. There is also a considerable amount of research on GABARAPL2. Studies have found that GABARAPL2 may be a gene related to mitophagy, and its expression was further validated through RT-qPCR and IHC, indicating that GABARAPL2 may be a prognostic biomarker and a candidate therapeutic target for GC (59). A study by Wang and colleagues revealed that GABARAPL2 may be a gene linked to autophagy. They also created a nomogram based on this gene to predict the prognosis of patients diagnosed with GC (60). Therefore, the role of GABARAPL2 in GC may be diverse, potentially involving multiple pathways leading to poor GC prognosis, and more research is needed to discover its specific mechanisms of action in order to bring real benefits to GC patients.

Our experimental results show that ANXA5 is significantly overexpressed in gastric cancer. When the expression of ANXA5 was suppressed via siRNA, we noted a reduction in cell viability, an increase in apoptosis, and diminished migratory and invasive abilities of gastric cancer cells. The results not only reinforce the role of ANXA5 as an oncogene in the development and progression of gastric cancer but also indicate that ANXA5 may act as a promising therapeutic target for the treatment of this cancer type. Furthermore, additional confirmation of these findings was provided by Western blot analysis, which indicated that the reduction of ANXA5 expression correlated with an increase in the pro-apoptotic protein c-Caspase-3 and the adhesion molecule E-Cadherin, along with a decrease in the anti-apoptotic protein Bcl-2 and the marker for epithelial-mesenchymal transition (EMT), Vimentin. The results indicate that ANXA5 might influence the behavior of gastric cancer cells by modifying the concentrations of vital proteins important for apoptosis and cell adhesion. In addition, ANXA5 has been shown to promote cancer in pancreatic, breast, and colorectal cancers (61–63). To sum up, our study underscores the crucial function of ANXA5 in gastric cancer progression, shedding light on the biological processes that drive this disease and presenting possible theoretical bases for upcoming treatment strategies.

In addition, although we have made efforts to predict the role of ANXA5 in GC through bioinformatics analysis and preliminary validation via *in vitro* experiments, further verification using patient-derived samples or tissue microarrays is warranted. Given the practical challenges in obtaining such samples, we acknowledge this as a limitation of our study and plan to incorporate human tissue validation in future research to strengthen our conclusions.

In summary, our study has made significant strides in understanding the cellular and molecular underpinnings of GC, with implications for diagnosis, prognosis, and treatment. While our findings are promising, further research is needed to validate these results in larger and more diverse patient populations and to translate these insights into clinical practice. The ongoing evolution of sequencing technologies and analytical methods will undoubtedly continue to enhance our ability to unravel the complexities of GC and other malignancies, bringing us closer to the goal of personalized and precise medicine.

## 5 Conclusion

In conclusion, this study’s comprehensive single-cell sequencing analysis not only elucidates the cellular heterogeneity in gastric adenocarcinoma but also establishes a clinically relevant risk signature that predicts patient prognosis and response to immunotherapy, underscoring the significance of single-cell technologies in advancing personalized medicine.

## Data Availability

The original contributions presented in the study are included in the article/supplementary material. Further inquiries can be directed to the corresponding authors.
